# Molecular genetic features and clinical manifestations in Chinese familial cerebral cavernous malformation: from a novel KRIT1/CCM1 mutation (c.1119dupT) to an overall view

**DOI:** 10.3389/fnins.2023.1184333

**Published:** 2023-05-05

**Authors:** Yanming Chen, Xuchen Dong, Ye Wang, Haijun Lv, Nan Chen, Zhongyong Wang, Si Chen, Ping Chen, Sheng Xiao, Jizong Zhao, Jun Dong

**Affiliations:** ^1^Department of Neurosurgery, Second Affiliated Hospital of Soochow University, Suzhou, China; ^2^Department of Neurosurgery, Huashan Hospital, Fudan University, Shanghai, China; ^3^Health Management Center, Second Affiliated Hospital of Soochow University, Suzhou, China; ^4^Department of Pathology, Second Affiliated Hospital of Soochow University, Suzhou, China; ^5^Suzhou Sano Precision Medicine Ltd., Suzhou, China; ^6^Department of Pathology, Brigham and Women’s Hospital, Boston, MA, United States; ^7^Department of Neurosurgery, Beijing Tiantan Hospital, Capital Medical University, Beijing, China; ^8^China National Clinical Research Center for Neurological Diseases, Beijing, China

**Keywords:** cerebral cavernous malformations (CCMs), Krev interaction trapped 1 (KRIT1), DNA sequencing, duplication mutation, frameshift

## Abstract

Cerebral cavernous malformations (CCMs) are common vascular anomaly diseases in the central nervous system associated with seizures, cerebral microbleeds, or asymptomatic mostly. CCMs can be classified as sporadic or familial, with familial cerebral cavernous malformations (fCCMs) being the autosomal dominant manner with incomplete penetrance. Germline mutations of KRIT1, CCM2, and PDCD10 are associated with the pathogenesis of fCCMs. Till now, little is known about the fCCMs mutation spectrum in the Han Chinese population. In this study, we enrolled a large, aggregated family, 11/26 of the family members were diagnosed with CCMs by pathological or neuroradiological examination, with a high percentage (5/9) of focal spinal cord involvement. Genomic DNA sequencing verified a novel duplication mutation (c.1119dupT, p.L374Sfs*9) in exon 9 of the Krev interaction trapped 1 (KRIT1) gene. The mutation causes a frameshift and is predicted to generate a truncated KRIT1/CCM1 protein of 381 amino acids. All our findings confirm that c.1119dupT mutation of KRIT1 is associated with fCCMs, which enriched the CCM genes’ mutational spectrum in the Chinese population and will be beneficial for deep insight into the pathogenesis of Chinese fCCMs. Additionally, with a retrospective study, we analyzed the molecular genetic features of Chinese fCCMs, most of the Chinese fCCMs variants are in the KRIT1 gene, and all these variants result in the functional deletion or insufficiency of the C-terminal FERM domain of the KRIT1 protein.

## Introduction

Cerebral cavernous malformations (CCMs) are vascular anomalies histologically characterized by tightly packed and abnormal capillary cavities collections, mainly located in the central nervous system (CNS) (Zafar et al., 2019). The clinical presentation of CCMs may be various, including seizures, recurrence headaches, focal neurological deficits, and hemorrhage (Ricci et al., 2021). However, many patients with CCMs are asymptomatic; hence, the real-world pre-valence of CCMs may be hard to accurately evaluate (Moore et al., 2014). CCMs most often occur sporadically or in familial form. Compared to sporadic CCMs usually present with CNS single lesions, fCCMs are characterized by CNS multiple lesions and autosomal dominant inheritance with incomplete penetrance (Riolo et al., 2021).

The pathogenic alterations of fCCMs have been mapped into three loci: KRIT1/CCM1 (OMIM 604214) (Dubovsky et al., 1995; Gunel et al., 1995), CCM2 (OMIM 607929) (Craig et al., 1998; Liquori et al., 2003), and PDCD10/CCM3 (OMIM 609118) (Bergametti et al., 2005), and are located on chromosomes 7q, 7p, and 3q, respectively. The incidence of fCCMs accounts for about 10−15% of all CCM cases (Zhao et al., 2011), and its incidence rate displays obvious geographical characteristics, with fCCMs incidence rates as high as 50% in Hispanic Americans and 10−40% in Caucasian populations (Rigamonti et al., 1988; Labauge et al., 2007). Epidemiologically, the incidence of fCCMs in Han Chinese has not been reported, systematic studies on Han Chinese fCCMs families are extremely rare, and clinically definite diagnosis of Han Chinese fCCMs is also rare (Yang et al., 2017; Liu et al., 2022). However, some Chinese-specific CCMs gene mutation loci have been found sporadically (Chen et al., 2002; Yang et al., 2017, 2018; Jih et al., 2018; Yang L. et al., 2020). Therefore, it is meaningful to uncover the genetic phenotype of Han Chinese fCCMs by continuously enriching the fCCMs’ genomic alteration spectrum in the Chinese population.

In this study, we report the clinical presentations, neuroradiological features, and genomic defects of a large Chinese fCCMs family. It’s beneficial for us to further characterize the clinical features of CCM patients with KRIT1 mutations and expand the mutational spectrum of this gene. Furthermore, a thorough retrospective study of molecular genetic features in Chinese fCCMs was conducted to gain a deeper insight into the etiology and pathogenic characteristics of Chinese fCCMs.

## Materials and methods

### Subjects

This study was approved by the ethics committee of the Second Affiliated Hospital of Soochow University. The family includes 26 members, 11/26 family members were diagnosed with the CNS CCMs, and two of them have departed. The clinical features of the 11 fCCMs patients have been shown in Table 1. The index patient (proband; II-1) is a 69-year-old male patient, suffering from progressive bilateral lower limbs dystonia. Over the next 6 years, the members of III-2, III-3, III-5, and IV-6 sequentially received surgeries and were confirmed to suffer the CNS CCMs. A retrospective study revealed that the family member of II-11 received surgery to remove an intracranial lesion due to seizures when he was young. The post-operative pathological diagnosis was “vascular malformation.” Unfortunately, no pathological or imaging sources were available, and without offspring. The family members of II-3, III-4, III-6, and IV-3 were all neuroradiological confirmations of CNS CCMs and were clinically silent.

**TABLE 1:** Clinical features and genetic data of the fCCMs members.

Items patients	Gender	Age at diagnosis (years)	Onset symptoms	Surgical treatment	Radiological location	Gene variant detection	Outcome
II-1	Male	69	Leg palsy, dysuria	Yes	Conus medullaris and multiple intracranial microscopic lesions	NGS and sanger sequencing	Alive
II-2	Male	45	Hemorrhage, conscious disturbance	Yes	–	–	Dead
II-3	Male	61	Asymptomatic	No	Right frontal, temporal and left temporal, occipital lobe multiple lesions	NGS and sanger sequencing	Alive
II-6	Female	16	Hemorrhage, Loss of consciousness	Yes	–	–	Dead
III-2	Female	47	Hemiparesis, headache	Yes	Pons, C5-6 cervical cord, left frontal lobe, and cerebellar hemisphere lesions	NGS and sanger sequencing	Alive
III-3	Male	34	Hemorrhage	Yes	Right frontal lobe, cervical and thoracic cord multiple lesions	Sanger sequencing	Alive
III-4	Female	38	Asymptomatic	No	Bilateral frontal lobe and cerebellar hemisphere lesions	Sanger sequencing	Alive
III-5	Female	40	Heemorrhage, seizure, and aphasia	Yes	Left frontal lobe, T5 thoracic cord and right thalamic lesions	Sanger sequencing	Alive
III-6	Male	45	Asymptomatic	No	Cerebellar hemisphere and left occipital lobe lesions	Sanger sequencing	Alive
IV-3	Male	24	Asymptomatic	No	Left frontal lobe and caudate, right frontal and occipital lobe lesions	NGS and sanger sequencing	Alive
IV-6	Female	16	Headache	Yes	Left frontal lobe, cervical and thoracic cord multiple minimal lesions	Sanger sequencing	Alive

### Whole-exome sequencing and causative variants filtering

We utilized whole-exome sequencing (WES) to detect potential pathogenic germline mutations within the fCCMs family members. Four fCCMs patient members (II-1, III-2, III-5, and IV-3) and two fCCMs health members (III-1 and III-7) were included in the WES study. The genomic DNA was extracted from each family member’s peripheral blood. DNA libraries were constructed and sequenced to a target depth of 100 × on the Illumina X-TEN platform, as previously reported (Han et al., 2020). Reads were aligned to the reference genome (hg19) using BWA-MEM. Sequencing results for single-nucleotide variations (SNVs) and insertion/deletion (Indels) were analyzed by GATK HaplotypeCaller.

The filtering process of candidate pathogenic variants: (1) Remove the variants with minor allele frequency (MAF) higher than 1% in the normal population database (gnomAD and 1000 Genomes); (2) The variants that occur in healthy Chinese and unaffected members of the family were removed; (3) Preference was given to variants that occur in both affected individuals and that are associated with cavernous malformations in the human genetic variation database ClinVar. Finally, manual identification of possible pathogenic variants, especially in the pathogenic genes KRIT1, CCM2, and PDCD10.

### Polymerase chain reaction (PCR) and sanger sequencing

PCR was performed with primers specific to KRIT1 (KRIT1-F: 5′-CCACTGAACTGTACGCCTAAA-3′, KRIT1-R: 5′-AAGTTGAGGCCACTCGCATAT-3′). 10 ng gDNA were used, and the PCR conditions were 95°C 5 min for 1 cycle followed by 35 cycles of 95°C 30 s, 60°C 30 s, and 72°C 1 min. A 424 bp PCR product was generated, which contained the candidate pathogenic mutation of KRIT1. The PCR product was analyzed by gel electrophoresis and directly Sanger sequenced.

### Three-dimensional structure analysis of KRIT1 protein

Currently, the most commonly used method for unknown protein structure prediction is based on the known protein structure. However, this method largely relies on homologous templates, which leads to the inability to predict protein structures without homologous templates. In this study, the Alphafold2 algorithm is used, which is based on a deep learning algorithm and can predict protein structures with atomic precision. It can still be predicted in the absence of homologous templates. As previously reported (Liang et al., 2022), using the default parameters of Alphafold2 to predict the structure of KRIT1 wild-type and mutant proteins, the optimal model was selected and obtained the protein data bank (PDB) files of the three-dimensional structure of KRIT1 protein. Meanwhile, to visualize the mutant and wild-type KRIT1 protein structures, the predicted PDB files were imported into Swiss-PDB Viewer (Version 4.0.4) for protein structure drawing.

## Results

### Clinical features

**FIGURE 1:**
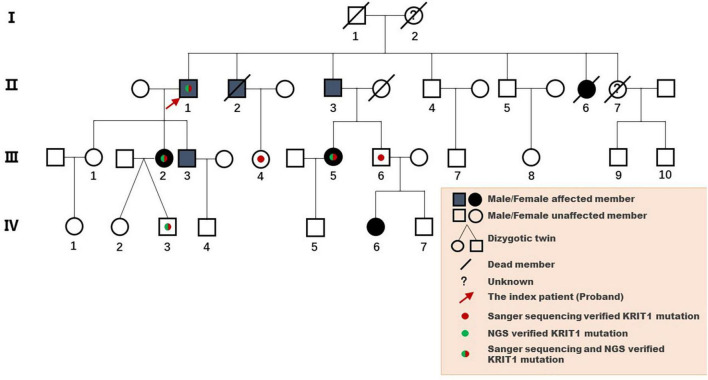
Pedigree diagram of the Chinese family with fCCMs. The affected family members were diagnosed with pathology or T2-weighted MRI of the CNS.

**FIGURE 2:**
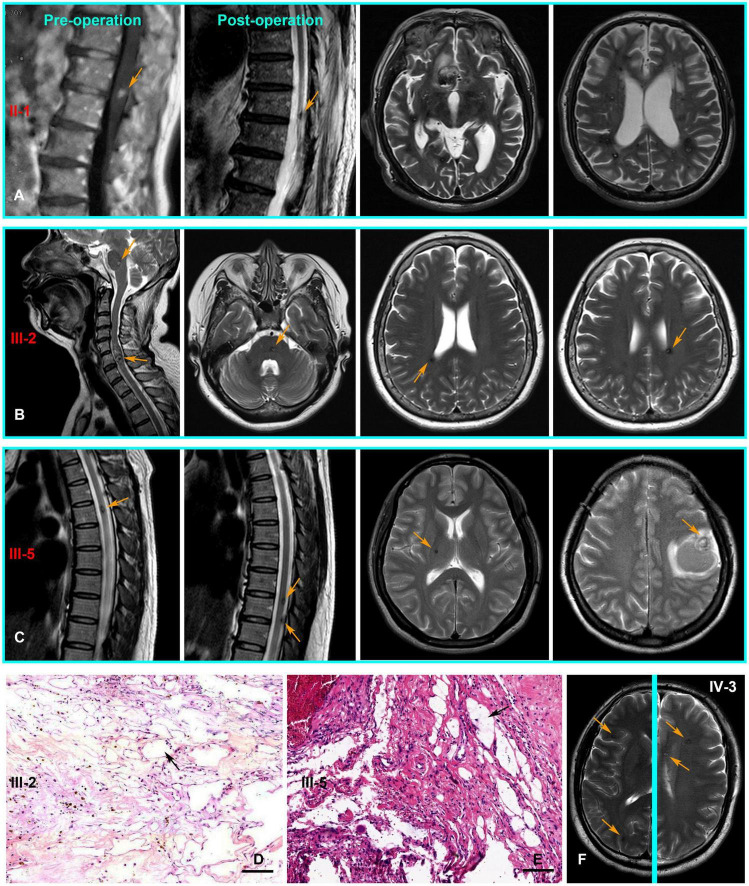
The CNS MRI and histopathology findings of patients with the CCM1 variant. **(A)** T1-weighted MR imaging of the proband (II-1) detected a lesion in the conus medullaris. Post-operative MR imaging revealed residual microfoci in the spinal cord, and the brain of T2-weighted MR imaging show multiple lesions of variable sizes in hemispheres. **(B,C)** Cerebral and spinal MR imaging showing there are multiple lesions in the CNS of III-3 and III-5, respectively. **(D,E)** Pathologically, hematoxylin and eosin (H and E, Scale bar = 100 μm) staining revealed vascular malformation associated with dilated vessels and thin-walled capillaries (black arrows), III-3 and III-5, respectively. **(F)** Multiple lesions were observed in an asymptomatic familial member (IV-3) with the KRIT1/CCM1 gene variant from T2-weighted MR imaging of different levels (Yellow arrows point to the lesions).

The pedigree was established with the help of family members (Figure 1). The index patient (proband; II-1) is a 69-year-old male patient. Spinal magnetic resonance imaging (MRI) of the T1-weighted imaging revealed a 1.0 cm diameter inhomogeneous hyperintense lesion in the conus medullaris (Figure 2A), and multiple intracranial mixed signal lesions of variable sizes were observed in the T2-weighted brain MR imaging (Figure 2A). Of the 11 fCCMs patient members, seven individuals were symptomatic, and four individuals were asymptomatic. The clinical presentation included paralysis (*n* = 2), headache (*n* = 2), loss of consciousness (*n* = 2), seizure (*n* = 1), and hemorrhage (*n* = 4) (Table 2). Seven patients underwent surgical treatment, and post-operative pathology verified the diagnosis of CCMs (Figures 2B-E). Four individuals neuroradiologically exhibited the CNS CCMs but were clinically asymptomatic (Figure 2F). Multiple lesions were found in 9/9 affected familial individuals (Figure 2). In this family of patients with CCMs, a high percentage (5/9) of patients had lesions involving the spinal cord (Table 2).

**TABLE 2:** Clinical features and radiological findings in the fCCMs patients.

Features	Total subjects (*N* = *11*)	Symptomatic subjects (*N* = *7*)	Asymptomatic subjects (*N* = *4*)
**Radiological findings**			
Multiple lesions	9	5	4
Single lesion	0	0	0
Brain involved	9	5	4
Spinal cord involved	5	5	0
**Onset symptoms**			
Paralysis	–	2	–
Headache	–	2	–
Loss of consciousness	–	2	–
Seizure	–	1	–
Hemorrhage	–	4	–
Others	–	2	–

### Molecular studies

**FIGURE 3:**
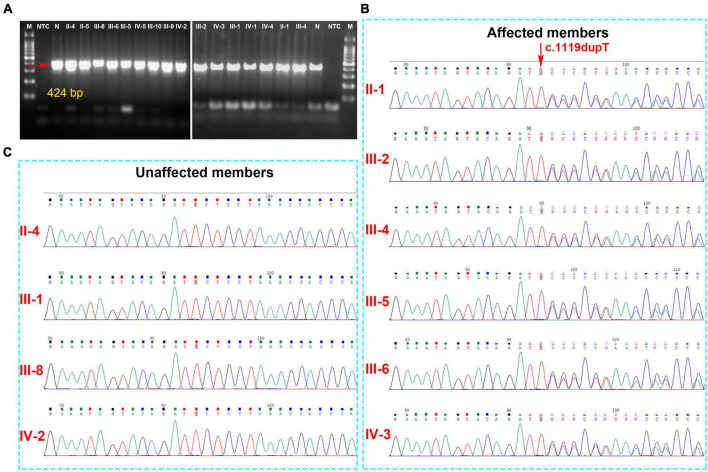
**(A)** The gel electrophoresis of PCR products shows KRIT1/CCM1 DNA segment containing the candidate mutant sites, and the PCR products were sequenced by the Sanger sequencing method. **(B)** A novel heterozygous duplication mutation (c.1119dupT, p.L374Sfs*9) of the KRIT1/CCM1 gene was detected by DNA sequencing in the CCM-affected members. The red arrow indicates a duplication of thymine at that site. **(C)** Representative sequencing results from the healthy family members indicate the absence of the KRIT1/CCM1 gene variant.

Analysis of the WES data found that four fCCMs individuals shared a common KRIT1/CCM1 (NM_004912) frameshift mutation (c.1119dupT, p.L374Sfs*9), while the candidate variation was exclusive in the two healthy familial individuals. The subsequent Sanger sequencing analysis of 16 familial individuals further confirmed that the candidate mutation of KRIT1/CCM1 was present in all members of the CCMs patients, while this mutation was unobserved in any of the ten healthy family members (Figure 3). This heterozygous duplication mutation (c.1119dupT) in exon 9 leads to a frameshift and is predicted to cause a premature termination codon to generate a truncated CCM1 protein of 381 amino acids. This mutation (c.1119dupT, p.L374Sfs*9) has never been reported in the following publicly available databases: CCM mutation database,^1^ NCBI single-nucleotide polymorphism (SNP),^2^ and the National Heart, Lung, and Blood Institute (NHLBI) Exome Sequencing Project Exome Variant Server.^3^

**FIGURE 4:**
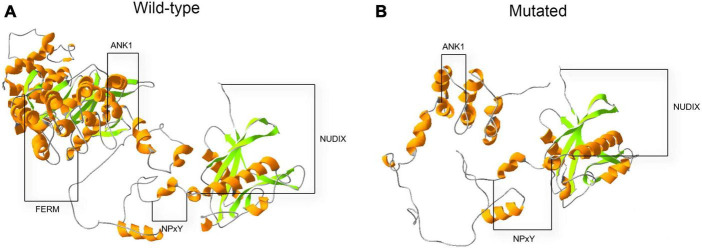
Three-dimensional structure prediction of wild-type and mutated KRIT1 proteins. The image compares native **(A)** and mutated **(B)** KRIT1 tertiary protein structures. As indicated, the main structural modifications affect ANK repeats domains at the C-terminal with consequent loss of the FERM domain.

An *in silico* prediction of the three-dimensional structures of wild-type and mutated KRIT1 proteins were performed based on Alphafold2. A structural comparison of the wild-type and mutated KRIT1 proteins indicated that ankyrin (ANK) repeats domains in the C-terminal were partially disrupted (Figure 4).

### Retrospective study

**FIGURE 5:**
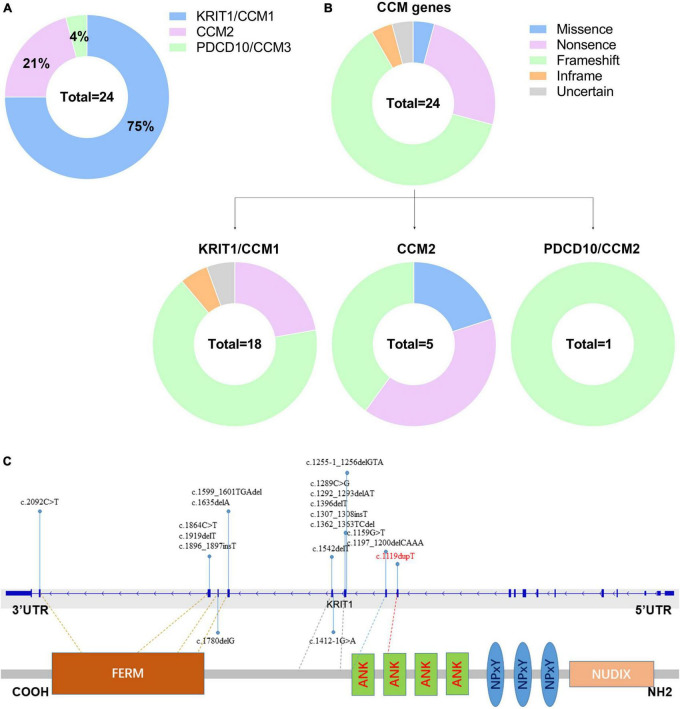
**(A)** Mutation spectrum of CCM genes in this cohort of Chinese fCCMs patients. **(B)** Distribution of different types of variants identified by each CCM gene, respectively. **(C)** Schematic representation of the gene structure and protein domains of KRIT1/CCM1 in the Chinese population. The upper panel represents the genetic structure, and the lower panel represents a corresponding protein product and its specific domain. Each reported KRIT1/CCM1 gene variant is marked in the corresponding genetic and amino acid position, respectively.

Till now, little is known referring to genetic alterations associated with fCCMs in the Chinese population. A retrieved result from Web of Science, PubMed, and Wanfang Data Knowledge Service Platform databases since 2000, with the terms familial cerebral cavernous malformations, mutation, and Chinese, articles published in English or Chinese with essential information about the mutations were included. We collected 24 pathogenetic alterations of fCCMs first identified in the Chinese population (Chen et al., 2002; Xu et al., 2003; Mao et al., 2005, 2016; Ji et al., 2006; Zhao et al., 2011; Wang et al., 2013, 2017, 2018; Zhu et al., 2014; Yang et al., 2017; Du et al., 2019; Han et al., 2020; Jiang et al., 2020; Yang L. et al., 2020; Yang Q. et al., 2020; Zhang et al., 2020; Liu et al., 2022). As exhibited in Table 3, the majority (18/24) of Chinese fCCMs pathogenetic alterations are in KRIT1/CCM1 gene. The incidence of CCM2 mutations followed, with only one Chinese fCCM carrying the PDCD10/CCM3 mutation (Figure 5A). Frameshift and Nonsense were the most common mutation types (Figure 5B), which always lead to a premature termination codon and cause to generate a truncated protein. In Chinese fCCMs carrying KRIT1/CCM1 variants, all these variants result in the functional deletion or insufficiency of the C-terminal FERM (a band four-point-one, ezrin, radixin, moesin) domain of the KRIT1 protein (Figure 5C). KRIT1 binds to microtubules, and when activated RAP1 binds to the FERM domain, KRIT1 translocates to adherens junctions and binds β-catenin (Francalanci et al., 2009; Nardella et al., 2018). However, as previously reported (Liu et al., 2011), the depletion of KRIT1-RAP1 interaction caused a deleterious decreased endothelial barrier function. CCM2 can interact with CCM1 via conserved NPxY motifs, and act as a bridge-like role in the CCM complex (Zawistowski et al., 2005; Draheim et al., 2015). Unlike Hispanic-American or Italian fCCMs (Ricci et al., 2021), no variants located at the three NPxY motifs were identified in this cohort of KRIT1/CCM1 variants of Chinese fCCMs.

**TABLE 3:** Summary of identified pathogenetic alterations of CCMs in the Chinese population.

No.	References	Gene symbols	Mutation sites (variant)	Mutation consequences	Protein
1	Chen et al., 2002	KRIT1/CCM1	c.2092C > T	Nonsense	p.Q698*
2	Xu et al., 2003	KRIT1/CCM1	c.1289C > G	Nonsense	p.S430*
3	Mao et al., 2005	KRIT1/CCM1	c.1292_1293delAT	Frameshift	p.Y431Sfs*4
4	Ji et al., 2006	KRIT1/CCM1	c.1255-1_1256delGTA	Frameshift	p.Q401Tfs*10
5	Zhao et al., 2011	KRIT1/CCM1	c.1197_1200delCAAA	Frameshift	p.Q401Tfs*10
6	Wang et al., 2013	KRIT1/CCM1	c.1396delT	Frameshift	p.C466Vfs*29
7	Zhu et al., 2014	KRIT1/CCM1	c.1542delT	Frameshift	p.L516Wfs*11
8	Mao et al., 2016	KRIT1/CCM1	c.1159G > T	Nonsense	p. E387*
9	Yang et al., 2017	KRIT1/CCM1	c.1780delG	Frameshift	p.A594Hfs*67
10	Yang et al., 2017	KRIT1/CCM1	c.1412-1G > A	Exon 14 skipping, or frameshift	p.S471Nfs*2 or p.S471Tfs*24
11	Wang et al., 2017	KRIT1/CCM1	c.1896_1897insT	Frameshift	p.P633Sfs*22
12	Yang et al., 2017	KRIT1/CCM1	c.1864C > T	Nonsense	p.Q622*
13	Yang et al., 2018	KRIT1/CCM1	c.1919delT	Frameshift	p.F640Sfs*21
14	Wang et al., 2018	KRIT1/CCM1	c.1599_1601TGAdel	Inframe	p.D533del
15	Wang et al., 2018	MGC4607/CCM2	c.773delA	Frameshift	p.K258fs*34
16	Du et al., 2019	MGC4607/CCM2	c.55C > T	Nonsense	p. R19*
17	Du et al., 2019	MGC4607/CCM2	c.*18G > A	Nonsense	Unknown?
18	Han et al., 2020	MGC4607/CCM2	c.331G > C	Missense	p.A111P
19	Jiang et al., 2020	PDCD10/CCM3	c.165delT	Frameshift	p.N55fs
20	Yang L. et al., 2020	MGC4607/CCM2	c.755delC	Frameshift	p.S252fs*40
21	Zhang et al., 2020	KRIT1/CCM1	c.1635delA	Frameshift	p.T545fs*6
22	Yang Q. et al., 2020	KRIT1/CCM1	c.1307_1308insT	Frameshift	p.L436Ffs*6
23	Liu et al., 2022	KRIT1/CCM1	c.1362_1363TCdel	Frameshift	p.Q455Kfs*23
24	Present	KRIT1/CCM1	c.1119dupT	Frameshift	p.L374Sfs*9

## Discussion

Since the three pathogenic gene loci (CCM1-3) were mapped, KRIT1/CCM1 mutations were disclosed in more than 40% of familial cases (Yang L. et al., 2020). As the most common pathogenetic mutational gene, more than 250 KRIT1/CCM1 mutations have been identified with a low degree of redundancy (Stenson et al., 2020). Noteworthily, all these mutations lead to premature stop codons that result in truncated proteins. However, pathogenic mutations of fCCMs exhibit remarkable genetic heterogeneity among different races and ethnicities. As a large ethnic population, the incidence of fCCMs is relatively lower among the Chinese population, and novel confirmed pathogenic mutations from the Chinese population are extremely rare. To date, little is known about the genomic alterations associated with fCCMs in the Chinese population. Hence, the identification of this novel CCM1 mutation expanded the mutational spectrum of CCM1 underlining familial CCMs.

In this study, we performed DNA sequencing to analyze a large Chinese family, with years of suffering from CNS cavernous malformations. We identified a novel pathogenic mutation of KRIT1/CCM1 in this family. This novel frameshift mutation of KRIT1 in exon 9 is predicted to cause a premature termination codon to generate a truncated KRIT1 protein of 381 amino acids. The truncated KRIT1 protein is speculated to suffer a severe structural and functional defect, which causes dysregulation of angiogenesis. Intriguingly, a high percentage (5/9) of lesions involving the spinal cord was observed in this family, which was rarely reported in previous studies.

Additionally, to deep insight into the molecular genomic alteration features of the Chinese fCCMs, we compiled the most comprehensive mutational spectrum of Chinese fCCMs to date. Our retrospective study indicated that KRIT1/CCM1 gene mutations were still the most common (18/24) pathogenic variants in the Chinese fCCMs, most of which result in the functional deletion or insufficiency of the C-terminal FERM domain of the KRIT1 protein. PDCD10/CCM3 mutations were extremely rare in this cohort of Chinese fCCMs. Consistent with previous reports, frameshift, and nonsense were the most common mutation types, which always lead to a premature termination codon and cause to generate a truncated protein. However, unlike Hispanic-American or Italian fCCMs, no variants were identified at the three NPxY motifs of KRIT1 protein in this cohort of Chinese fCCMs.

Emerging evidence indicated that fCCMs might exhibit great genetic heterogeneity among different geographic ethnicities. Till now, little is known regarding germline mutations leading to fCCMs in the Chinese population. In this study, we described a novel mutation in KRIT1/CCM1 gene, and retrospectively analyzed the scarcity and sporadic reports of germline mutations of fCCMs in the Chinese population. The population-specific mutational analysis of fCCMs is of a warrant to deep insight into the mutational spectrum of the Chinese population.

## Conclusion

We identified a novel germline mutation (c.1119dupT) of KRIT1/CCM1 from a Chinese fCCMs family, which enriches the fCCMs pathogenic mutational spectrum in the Chinese population. The family member carrying this novel CCM1 variant suffered approximately 100% neuroradiological penetrance, which will be beneficial for prenatal guidance and genetic counseling. Moreover, a thorough retrospective study of molecular genetic features in Chinese fCCMs facilitates us to gain a deeper insight into the etiology and pathogenic characteristics of Chinese fCCMs.

## Data availability statement

The original contributions presented in this study are publicly available. This data can be found here: https://www.ncbi.nlm.nih.gov/sra/PRJNA958149.

## Ethics statement

The studies involving human participants were reviewed and approved by the Ethics Committee of the Second Affiliated Hospital of Soochow University. The patients/participants provided their written informed consent to participate in this study. Written informed consent was obtained from the individual(s) for the publication of any potentially identifiable images or data included in this article.

## Author contributions

YC designed this study. XD and HL were in charge of pathological diagnosis. YW, ZW, and PC supported and analyzed clinical data. NC and SC supported the genomic analysis. SX, JZ, and JD guided this work and reviewed the manuscript. All authors approved the final version of the submitted manuscript.

## Footnotes

1
http://www.angiomaalliance.org

2
http://www.ncbi.nlm.nih.gov/snp

3
http://evs.gs.washington.edu/EVS/

## References

[B1] Bergametti F. Denier C. Labauge P. Arnoult M. Boetto S. Clanet M. (2005). Mutations within the programmed cell death 10 gene cause cerebral cavernous malformations. *Am. J. Hum. Genet.* 76 42–51. 15543491 10.1086/426952 PMC1196432

[B2] Chen D. Lipe H. Qin Z. Bird T. (2002). Cerebral cavernous malformation: novel mutation in a Chinese family and evidence for heterogeneity. *J. Neurol. Sci.* 196 91–96. 10.1016/s0022-510x(02)00031-x 11959162

[B3] Craig H. Gunel M. Cepeda O. Johnson E. Ptacek L. Steinberg G. (1998). Multilocus linkage identifies two new loci for a mendelian form of stroke, cerebral cavernous malformation, at 7p15-13 and 3q25.2-27. *Hum. Mol. Genet.* 7 1851–1858. 10.1093/hmg/7.12.1851 9811928

[B4] Draheim K. Li X. Zhang R. Fisher O. Villari G. Boggon T. (2015). CCM2-CCM3 interaction stabilizes their protein expression and permits endothelial network formation. *J. Cell Biol.* 208 987–1001. 10.1083/jcb.201407129 25825518 PMC4384732

[B5] Du Q. Shi Z. Chen H. Zhang Y. Wang J. Zhou H. (2019). Two novel CCM2 heterozygous mutations associated with cerebral cavernous malformation in a Chinese family. *J. Mol. Neurosci.* 67 467–471. 10.1007/s12031-018-1254-4 30701383

[B6] Dubovsky J. Zabramski J. Kurth J. Spetzler R. Rich S. Orr H. (1995). A gene responsible for cavernous malformations of the brain maps to chromosome 7q. *Hum. Mol. Genet.* 4 453–458. 10.1093/hmg/4.3.453 7795602

[B7] Francalanci F. Avolio M. De Luca E. Longo D. Menchise V. Guazzi P. (2009). Structural and functional differences between KRIT1A and KRIT1B isoforms: a framework for understanding CCM pathogenesis. *Exp. Cell Res.* 315 285–303. 10.1016/j.yexcr.2008.10.006 18992740

[B8] Gunel M. Awad I. Anson J. Lifton R. (1995). Mapping a gene causing cerebral cavernous malformation to 7q11.2-q21. *Proc. Natl. Acad. Sci. U.S.A.* 92 6620–6624. 7604043 10.1073/pnas.92.14.6620 PMC41570

[B9] Han G. Ma L. Qiao H. Han L. Wu Q. Li Q. (2020). A novel CCM2 missense variant caused cerebral cavernous malformations in a Chinese family. *Front. Neurosci.* 14:604350. 10.3389/fnins.2020.604350 33469417 PMC7813800

[B10] Ji B. Qin W. Sun T. Feng G. He L. Wang Y. J. (2006). A novel deletion mutation in CCM1 gene (krit1) is detected in a Chinese family with cerebral cavernous malformations. *Yi Chuan Xue Bao.* 33 105–110. 10.1016/S0379-4172(06)60028-0 16529293

[B11] Jiang X. Zhang Y. Yin X. Nan D. Wang X. Feng J. (2020). A novel CCM3 mutation associated with cerebral cavernous malformation in a Chinese family. *Ther. Adv. Neurol. Dis.* 13:1756286420902664. 10.1177/1756286420902664 32071616 PMC6997961

[B12] Jih K. Chung C. Chang Y. Hung P. Soong B. Liao Y. (2018). Mutational analysis of CCM1, CCM2 and CCM3 in a han Chinese cohort with multiple cerebral cavernous malformations in Taiwan. *Clin. Genet.* 94 389–390. 10.1111/cge.13377 29787619

[B13] Labauge P. Denier C. Bergametti F. Tournier-Lasserve E. (2007). Genetics of cavernous angiomas. *Lancet Neurol.* 6 237–244. 17303530 10.1016/S1474-4422(07)70053-4

[B14] Liang X. Zhang H. Wang Z. Zhang X. Dai Z. Zhang J. (2022). JMJD8 Is an M2 macrophage biomarker, and it associates with DNA damage repair to facilitate stemness maintenance, chemoresistance, and immunosuppression in pan-cancer. *Front. Immunol.* 13:875786. 10.3389/fimmu.2022.875786 35898493 PMC9309472

[B15] Liquori C. Berg M. Siegel A. Huang E. Zawistowski J. Stoffer T. (2003). Mutations in a gene encoding a novel protein containing a phosphotyrosine-binding domain cause type 2 cerebral cavernous malformations. *Am. J. Hum. Genet.* 73 1459–1464. 10.1086/380314 14624391 PMC1180409

[B16] Liu J. Stockton R. Gingras A. Ablooglu A. Han J. Bobkov A. (2011). A mechanism of Rap1-induced stabilization of endothelial cell–cell junctions. *Mol. Biol. Cell.* 22 2509–2519. 10.1091/mbc.E11-02-0157 21633110 PMC3135476

[B17] Liu W. Liu M. Lu D. Wang J. Cao Z. Liu X. (2022). A Chinese Family with cerebral cavernous malformation caused by a frameshift mutation of the CCM1 gene: a case report and review of the literature. *Front. Neurol.* 13:795514. 10.3389/fneur.2022.795514 35444609 PMC9013744

[B18] Mao C. Yang J. Zhang S. Luo H. Song B. Liu Y. (2016). Exome capture sequencing identifies a novel CCM1 mutation in a Chinese family with multiple cerebral cavernous malformations. *Int. J. Neurosci.* 126 1071–1076. 10.3109/00207454.2015.1118628 26643368

[B19] Mao Y. Zhao Y. Zhou L. Huang C. Shou X. Gong J. (2005). A novel gene mutation (1292 deletion) in a Chinese family with cerebral cavernous malformations. *Neurosurgery* 56 1149–1153. 15854263

[B20] Moore S. Brown R. Jr. Christianson T. Flemming K. (2014). Long-term natural history of incidentally discovered cavernous malformations in a single-center cohort. *J. Neurosurg.* 120 1188–1192. 10.3171/2014.1.JNS131619 24628608

[B21] Nardella G. Visci G. Guarnieri V. Castellana S. Biagini T. Bisceglia L. (2018). A single-center study on 140 patients with cerebral cavernous malformations: 28 new pathogenic variants and functional characterization of a PDCD10 large deletion. *Hum. Mutat.* 39 1885–1900. 10.1002/humu.23629 30161288

[B22] Ricci C. Cerase A. Riolo G. Manasse G. Battistini S. (2021). KRIT1 gene in patients with cerebral cavernous malformations: clinical features and molecular characterization of novel variants. *J. Mol. Neurosci.* 71 1876–1883. 10.1007/s12031-021-01814-w 33651268 PMC8421287

[B23] Rigamonti D. Hadley M. Drayer B. Johnson P. Hoenig-Rigamonti K. Knight J. (1988). Cerebral cavernous malformations. Incidence and familial occurrence. *N. Engl. J. Med.* 319 343–347. 3393196 10.1056/NEJM198808113190605

[B24] Riolo G. Ricci C. Battistini S. (2021). Molecular genetic features of cerebral cavernous malformations (CCM) patients: an overall view from genes to endothelial cells. *Cells* 10:3. 10.3390/cells10030704 33810005 PMC8005105

[B25] Stenson P. Mort M. Ball E. Chapman M. Evans K. Azevedo L. (2020). The Human gene mutation database (HGMD((R))): optimizing its use in a clinical diagnostic or research setting. *Hum. Genet.* 139 1197–1207. 32596782 10.1007/s00439-020-02199-3 PMC7497289

[B26] Wang H. Pan Y. Zhang Z. Li X. Xu Z. Suo Y. (2017). A novel KRIT1/CCM1 gene insertion mutation associated with cerebral cavernous malformations in a Chinese family. *J. Mol. Neurosci.* 61 221–226. 10.1007/s12031-017-0881-5 28160210

[B27] Wang K. Wu D. Zhang B. Zhao G. (2018). Novel KRIT1/CCM1 and MGC4607/CCM2 gene variants in Chinese families with cerebral cavernous malformations. *Front. Neurol.* 9:1128. 10.3389/fneur.2018.01128 30622508 PMC6308150

[B28] Wang X. Liu X. Lee N. Liu Q. Li W. Han T. (2013). Features of a Chinese family with cerebral cavernous malformation induced by a novel CCM1 gene mutation. *Chin. Med. J.* 126 3427–3432. 24034083

[B29] Xu Y. Zhao J. Wu B. Zhong H. Wang S. Heng W. (2003). [A novel Krit-1 mutation in han family with cerebral cavernous malformation]. *Zhonghua Bing Li Xue Za Zhi* 32 220–225. 12882686

[B30] Yang C. Wu B. Zhong H. Li Y. Zheng X. Xu Y. (2018). A novel CCM1/KRIT1 heterozygous deletion mutation (c.1919delT) in a Chinese family with familial cerebral cavernous malformation. *Clin. Neurol. Neurosurg.* 164 44–46. 10.1016/j.clineuro.2017.11.005 29169046

[B31] Yang C. Zhao J. Wu B. Zhong H. Li Y. Xu Y. (2017). Identification of a novel deletion mutation (c.1780delG) and a novel splice-site mutation (c.1412-1G>A) in the CCM1/KRIT1 gene associated with familial cerebral cavernous malformation in the chinese population. *J. Mol. Neurosci.* 61 8–15. 10.1007/s12031-016-0836-2 27649701

[B32] Yang L. Wu J. Zhang J. (2020). A novel CCM2 gene mutation associated with cerebral cavernous malformation. *Front. Neurol.* 11:70. 10.3389/fneur.2020.00070 32117029 PMC7020567

[B33] Yang Q. Liu H. Guo J. Liu Q. Dou W. (2020). Case report of familial cerebral cavernous hemangioma with new mutation site and literature review. *Neural Injury Funct. Reconstruct.* 15 497–500.

[B34] Zafar A. Quadri S. Farooqui M. Ikram A. Robinson M. Hart B. (2019). Familial cerebral cavernous malformations. *Stroke* 50 1294–1301. 30909834 10.1161/STROKEAHA.118.022314 PMC6924279

[B35] Zawistowski J. Stalheim L. Uhlik M. Abell A. Ancrile B. Johnson G. (2005). CCM1 and CCM2 protein interactions in cell signaling: implications for cerebral cavernous malformations pathogenesis. *Hum. Mol. Genet.* 14 2521–2531. 16037064 10.1093/hmg/ddi256

[B36] Zhang F. Xue Y. Zhang F. Wei X. Zhou Z. Ma Z. (2020). Identification of a novel CCM1 frameshift mutation in a Chinese han family with multiple cerebral cavernous malformations. *Front. Neurosci.* 14:525986. 10.3389/fnins.2020.525986 33071727 PMC7538688

[B37] Zhao Y. Xie L. Li P. Song J. Qu T. Fan W. (2011). A novel CCM1 gene mutation causes cerebral cavernous malformation in a Chinese family. *J. Clin. Neurosci.* 18 61–65. 10.1016/j.jocn.2010.04.051 20884211

[B38] Zhu H. Guo Y. Feng X. Zhang R. Zhou C. Li G. (2014). Familial cerebral cavernous angiomas: clinical and genetic features in a Chinese family with a frame-shift mutation in the CCM1 gene (krit1). *J. Mol. Neurosci.* 54 790–795. 10.1007/s12031-014-0415-3 25185960

